# High fat diets induce early changes in gut microbiota that may serve as markers of ulterior altered physiological and biochemical parameters related to metabolic syndrome. Effect of virgin olive oil in comparison to butter

**DOI:** 10.1371/journal.pone.0271634

**Published:** 2022-08-16

**Authors:** Natalia Andújar-Tenorio, Isabel Prieto, Antonio Cobo, Ana M. Martínez-Rodríguez, Marina Hidalgo, Ana Belén Segarra, Manuel Ramírez, Antonio Gálvez, Magdalena Martínez-Cañamero

**Affiliations:** 1 Área de Microbiología, Departamento de Ciencias de la Salud, Universidad de Jaén, Jaén, Spain; 2 Área de Fisiología, Departamento de Ciencias de la Salud, Universidad de Jaén, Jaén, Spain; 3 Departamento de Estadística e Investigación Operativa, Universidad de Jaén, Jaén, Spain; Institute for Biological Research, University of Belgrade, SERBIA

## Abstract

Butter and virgin olive oil (EVOO) are two fats differing in their degree of saturation and insaponifiable fraction. EVOO, enriched in polyphenols and other minority components, exerts a distinct effect on health. Using next generation sequencing, we have studied early and long-term effects of both types of fats on the intestinal microbiota of mice, finding significant differences between the two diets in the percentage of certain bacterial taxa, correlating with hormonal, physiological and metabolic parameters in the host. These correlations are not only concomitant, but most noticeably some of the changes detected in the microbial percentages at six weeks are correlating with changes in physiological values detected later, at twelve weeks. *Desulfovibrionaceae*/*Desulfovibrio*/*D*. *sulfuricans* stand out by presenting at six weeks a statistically significant higher percentage in the butter-fed mice with respect to the EVOO group, correlating with systolic blood pressure, food intake, water intake and insulin at twelve weeks. This not only suggests an early implication in the probability of developing altered physiological and biochemical responses later on in the host lifespan, but also opens the possibility of using this genus as a marker in the risk of suffering different pathologies in the future.

## Introduction

The type of diet consumed has an influence on the intestinal microbiota [1]. In particular, high fat diets have a marked impact on the host microbial community [2–4] and have been extensively studied due to their negative effects on health, promoting pathologic conditions such as, for instance, metabolic syndrome [2, 5]. The effects of lipids on health depend on their degree of saturation [6–8]. Diets with high percentage of unsaturated fatty acids have been associated with beneficial effects such as a lower incidence of metabolic syndrome [9], and extra virgin olive oil (EVOO), a monounsaturated fat, is considered to be a healthy fat during the treatment of chronic inflammation on different diseases [10, 11]. Moreover, EVOO has been widely studied for being the main fat in the Mediterranean Diet and for its appreciated sensorial characteristics [9]. Beneficial properties of EVOO are not only attributed to the fatty acid composition, but also to the unsaponifiable matter as it contains high diversity of polyphenols [12–16]. Polyphenols are well known for their antioxidant effects [17] and antimicrobial activity on certain types of bacteria [18].

Our recent studies have shown the effects of different types of fats on mouse intestinal microbiota as well as on several physiological and biochemical parameters. We included butter as an example of saturated fat, and olive oil, as unsaturated, and their effects were compared using genotyping methods [13] and massive sequencing [19, 20]. Evidences supporting a link between specific types of diets, certain bacterial taxa and physiological parameters related to metabolic syndrome were uncovered after 12 weeks of diet. The results demonstrated a direct correlation between several variables involved in metabolic syndrome (insulin, diuresis, blood pressure, body weight) measured at 12 weeks, with bacterial taxa that at that time point were significantly increased under a butter enriched diet (BT) and decreased with an EVOO enriched diet [19]. The influence of these types of fat on the microbiota was also confirmed by culture-dependent methods, when a collection of enterococcal strains was isolated from faeces of mice after the diet intervention [21, 22].

All these data indicate that the positive effects of EVOO on health are significantly accompanied by parallel changes on the gut microbiota. However, correlations do not necessarily mean causality, as variations on gut microbiota could be the consequence of changes in physiological parameters caused by olive oil and not the other way around. With the purpose of adding more information in this sense, we have studied the intestinal taxa and the physiological variables of the host in the previous weeks of our former experiment, that is after six weeks of the initiation of diet, in order to find data that help us determine the causality in these relationships. A deep analysis provided strongly interesting results that we report here: how early changes in the prevalence of certain intestinal bacterial taxa correlate with specific later changes in hormonal, physiological and biochemical parameters.

## Results

### Physiological parameters

Table 1 shows the data obtained in the physiological variables after six weeks of the experimental period. The only clear difference found was in the systolic blood pressure, where the EVOO diet showed significant lower values than the standard (SD) and BT diets (p = 0.004). Weight was also non-significantly lower in EVOO mice and higher in BT, marking a good tendency (p = 0.148). However, values in the rest of variables measured (food intake, water intake and diuresis) were very similar between diets (p = 0.87, 0.70, 0.66, respectively).

**Table 1:** Physiological responses of mice after six weeks of diet.

	SD	EVOO	BT	p
**Food Intake (g/day)**	3.75±0.39	4.06±0.51	4.08±0.50	n.s.
**Water intake (g/day)**	9.27±0.76	9.28±1.24	10.4±1.14	n.s.
**Diuresis (mL/day)**	1.77±0.60	2.24±0.55	1.67±0.34	n.s.
**Body Weight (g)**	41.13±1.60	39.01±0.79	42.3±1.11	n.s.
**Systolic Blood Pressure (mmHg)**	164.75±6.4	134.3±2.77	165.67±12.14	a

a: Differences in extra virgin olive oil (EVOO) *vs* standard (SD) and butter (BT) diets, p<0.01.

### Sequencing, taxa adscription, percentage comparison and correlations

The number of reads was stable after pyrosequencing the 26 samples and, after trimming and filtering, a mean length between 549 and 568 nt and a total amount of 148.16 MB was obtained. A blast search was performed in order to associate an organism to each sequence obtained. Once we had the associations, the reads were filtered, analysed and grouped based on family, genera and species levels. In total, 268315 sequences were detected and classified into 8 phyla, 88 families, 227 genera and 538 species.

**Fig 1:**
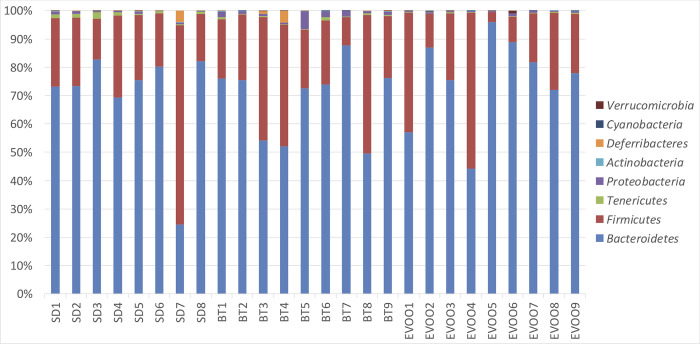
Global bacterial phyla distribution for each diet as a percentage of total sequences after 6 weeks of treatment. Each column corresponds to one animal fed a diet enriched with butter (BT), virgin olive oil (EVOO) or standard chow (SD). Legend shows upwards the eight phyla detected.

**Fig 2:**
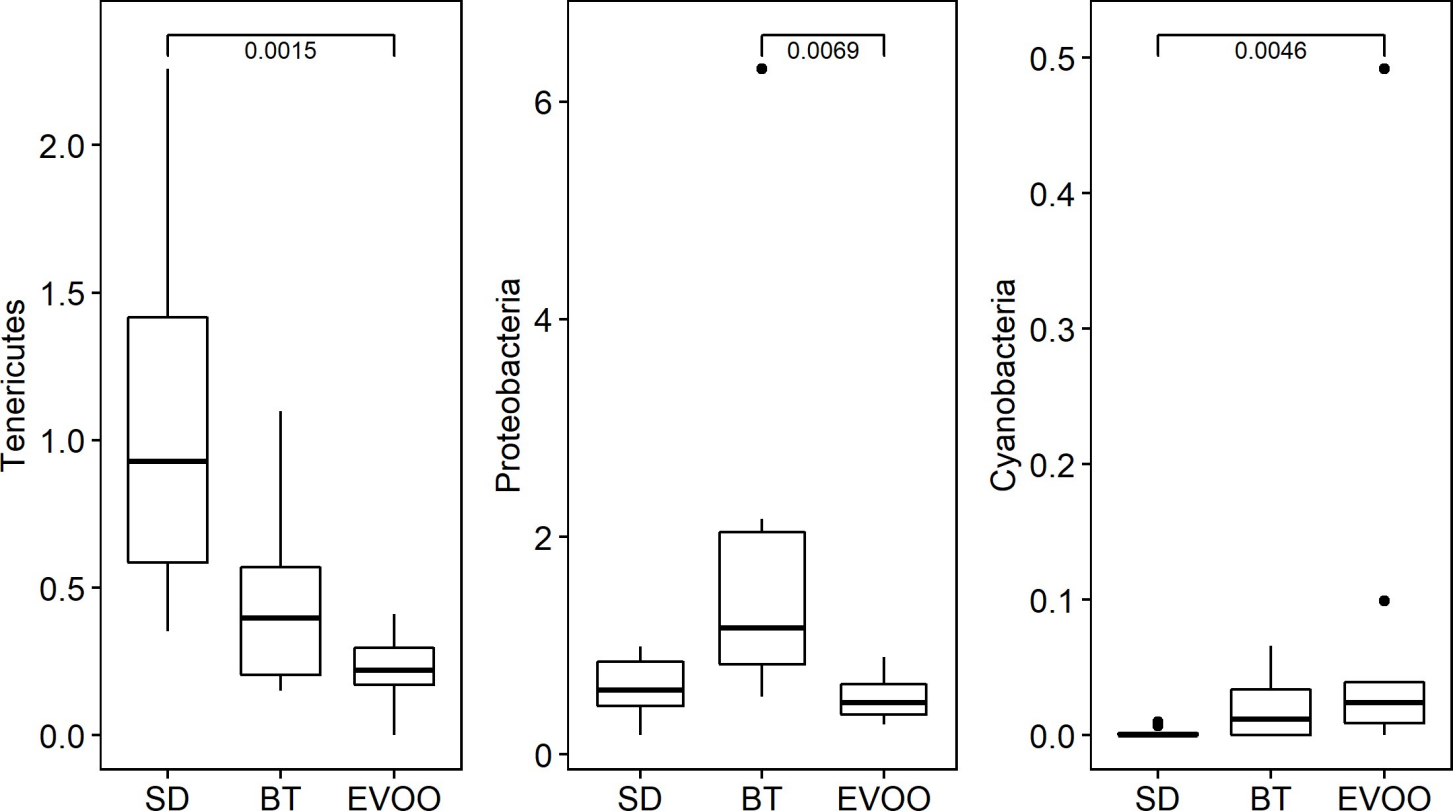
Box-plot representation of the percentage distribution of phyla *Tenericutes*, *Proteobacteria* and *Cyanobacteria* in mice fed a diet enriched with butter (BT), virgin olive oil (EVOO) or standard chow (SD).

Kruskal-Wallis test was used to check if the distributions of the diverse phyla were the same among the three diets (Fig 1). The phyla *Tenericutes* and *Proteobacteria* showed significant differences (p = 0.008 and p = 0.006 respectively). Pairwise comparisons were performed showing that there are significant differences, being EVOO values lower than those of SD in the first case and those of BT in the second case. *Cyanobacteria* also showed significant differences (p = 0.02) with greater values in EVOO versus SD, however it is important to remark the high number of data points with a value of 0 in this phylum. Fig 2 shows the box-plot of the distribution of these phyla in the three diets. A linear regression analysis was performed for each physiological variable considering the phyla as independent variables. Since we had also available the physiological measurements at the end of the experiment, already reported in Prieto et al., 2018 [19], we repeated the regression analysis with these variables as dependent. The results obtained are showed in Table 2. Conforming to this, the phylum *Proteobacteria* showed negative correlation with blood pressure at 6 weeks (R^2^ = 0.69; p = 0.0028) but, together with *Tenericutes*, also showed a positive relation with glucose at 12 weeks (R^2^ = 0.60; p = 0.0207).

**Table 2:** Regression fits for each of the physiological variables studied using as independent variables those phyla that show statistical differences in the percentage of sequences retrieved from faecal samples.

	SBP	Glu	Leptin*
	**6w**	**12w**	**12w**
	0.69 0.0028	0.60 0.0207	0.18 0.039
*Tenericutes*		1213.20 ±441.65 (0.0138)	
*Proteobacteria*	-20.61 ±6.69 (0.0081)	1230.34 ±442.42 (0.0128)	
*Cyanobacteria*			-2.55 ±1.16 (0.0389)

For each case, regression coefficient estimate, s.e. and p values are shown. R^2^ and p values of the model are also indicated under each physiological variable.

* indicates that logarithms of data have been used for the analysis. FI, food intake; WI, water intake; BW, body weight; SBP, systolic blood pressure; T-CHO, total cholesterol.

**Fig 3:**
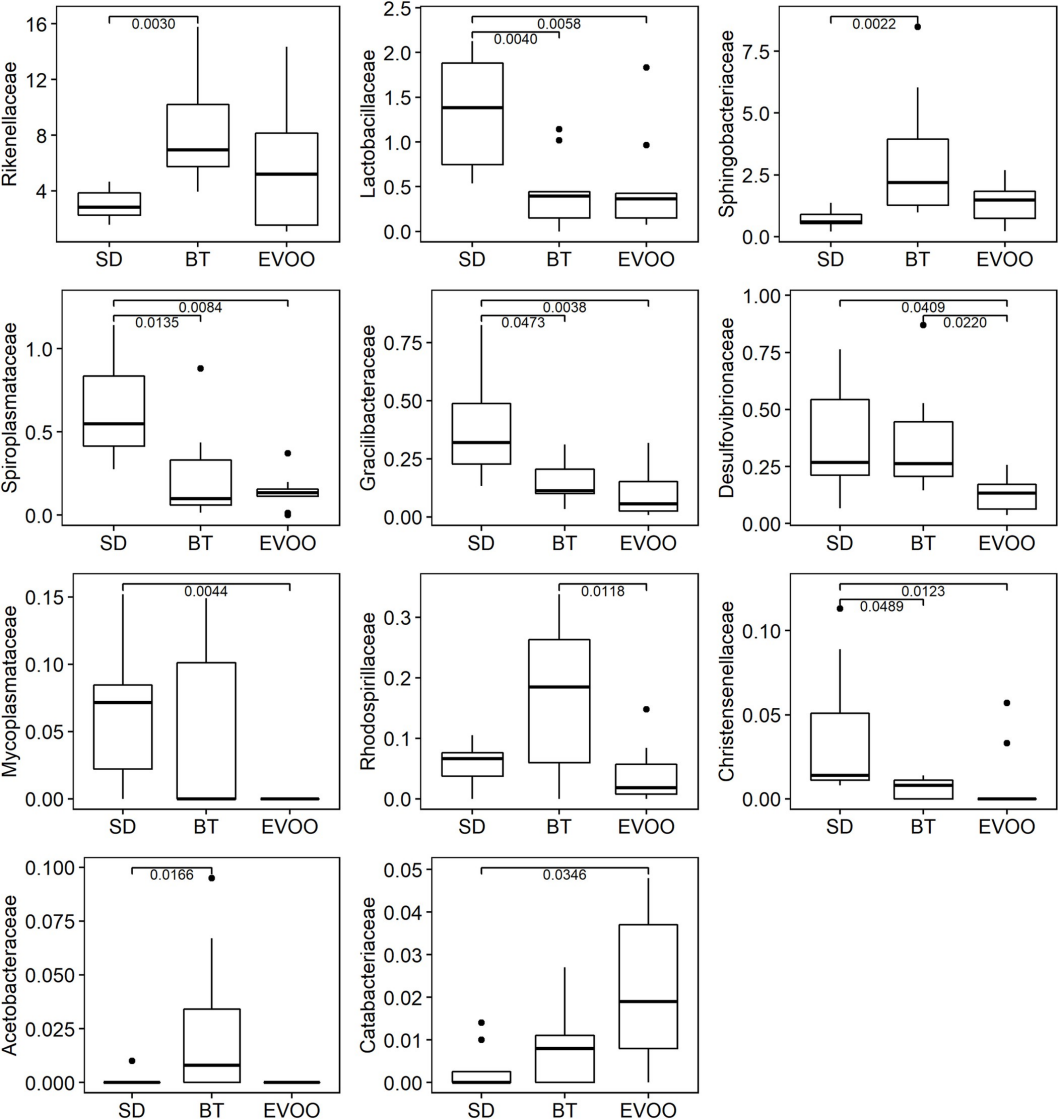
Box-plot representation of the percentage distribution of the eleven families with significant differences (p<0.05) among mice fed a diet enriched with butter (BT), virgin olive oil (EVOO) or standard chow (SD).

According to the Kruskal-Wallis test, out of the 88 families that were detected, only eleven of them showed statistically significant differences among the diets. Fig 3 displays the box-plots of these eleven families with their pairwise comparisons. Different multiple linear regression models were fitted to explain each physiological variable (both at six and 12 weeks) using as independent variables all the families with significant differences (Table 3).

**Table 3:** Regression fits for each of the physiological variables studied using as independent variables those families that show statistical differences in the percentage of sequences retrieved from faecal samples.

	BW		FI		WI		DIU		SBP		HDL/LDL	Insulin
	6w	12w	6w	12w*	6w	12w	6w*	12w*	6w	12w	12w*	12w*
	0.52 0.0002	0.50 0.0162	0.51 0.0037	0.68 0.0003	0.19 0.0374	0.19 0.0404	0.72 0.0071	0.42 0.0376	0.24 0.0281	0.75 0.0001	0.69 0.0001	0.21 0.0200
*Rikenellaceae*		-0.46 ±0.18 (0.0165)	0.17 ±0.05 (0.0072)	0.04 ±0.01 (0.0331)	0.32 ±0.14 (0.0374)		0.27 ±0.05 (0.0002)				0.10 ±0.02 (0.0000)	
*Lactobacillaceae*			1.34 ±0.40 (0.0029)	0.34 ±0.13 (0.0196)								
*Sphingobacteriaceae*		1.49 ±0.45 (0.0037)					-0.51 ±0.13 (0.0017)	-0.23 ±0.09 (0.0211)		10.53 ±2.53 (0.0006)		
*Spiroplasmataceae*		5.90 ±2.02 (0.0089)							34.93 ±14.62 (0.0281)	30.96 ±13.43 (0.0333)		
*Gracillibacteraceae*	9.63 ±3.69 (0.0016)		-3.46 ±1.28 (0.0132)	-1.33 ±0.45 (0.0081)				-1.54 ±0.68 (0.0380)				
*Desulfovibrionaceae*										41.65 ±17.45 (0.0281)	0.80 ±0.31 (0.0185)	1.36 ±0.55 (0.0200)
*Mycoplasmataceae*		-35.79 ±14.01 (0.0194)					17.14 ±4.07 (0.0010)			-193.50 ±85.86 (0.0369)		
*Rhodospirillaceae*							7.84 ±2.09 (0.0025)	5.02 ±1.83 (0.0152)			-1.69 ±0.68 (0.0214)	
*Christensenellaceae*				6.24 ±2.61 (0.0272)			17.24 ±5.96 (0.0125)					
*Acetobacteraceae*	86.94 ±22.45 (0.0008)	59.86 ±22.72 (0.0163)	-20.21 ±9.50 (0.0454)	-14.20 ±2.83 (0.0001)		-84.99 ±38.79 (0.0405)	-19.20 ±8.77 (0.0474)			606.01 ±173.30 (0.0025)		
*Catabacteriaceae*							54.54 ±13.05 (0.0011)				15.87 ±4.67 (0.0029)	

For each case, regression coefficient estimate, s.e. and p values are shown. R^2^ and p values of the model are also indicated under each physiological variable.

* indicates that logarithms of data have been used for the analysis. BW, body weight; FI, food intake; WI, water intake; DIU, diuresis; SBP, systolic blood pressure.

**Fig 4:**
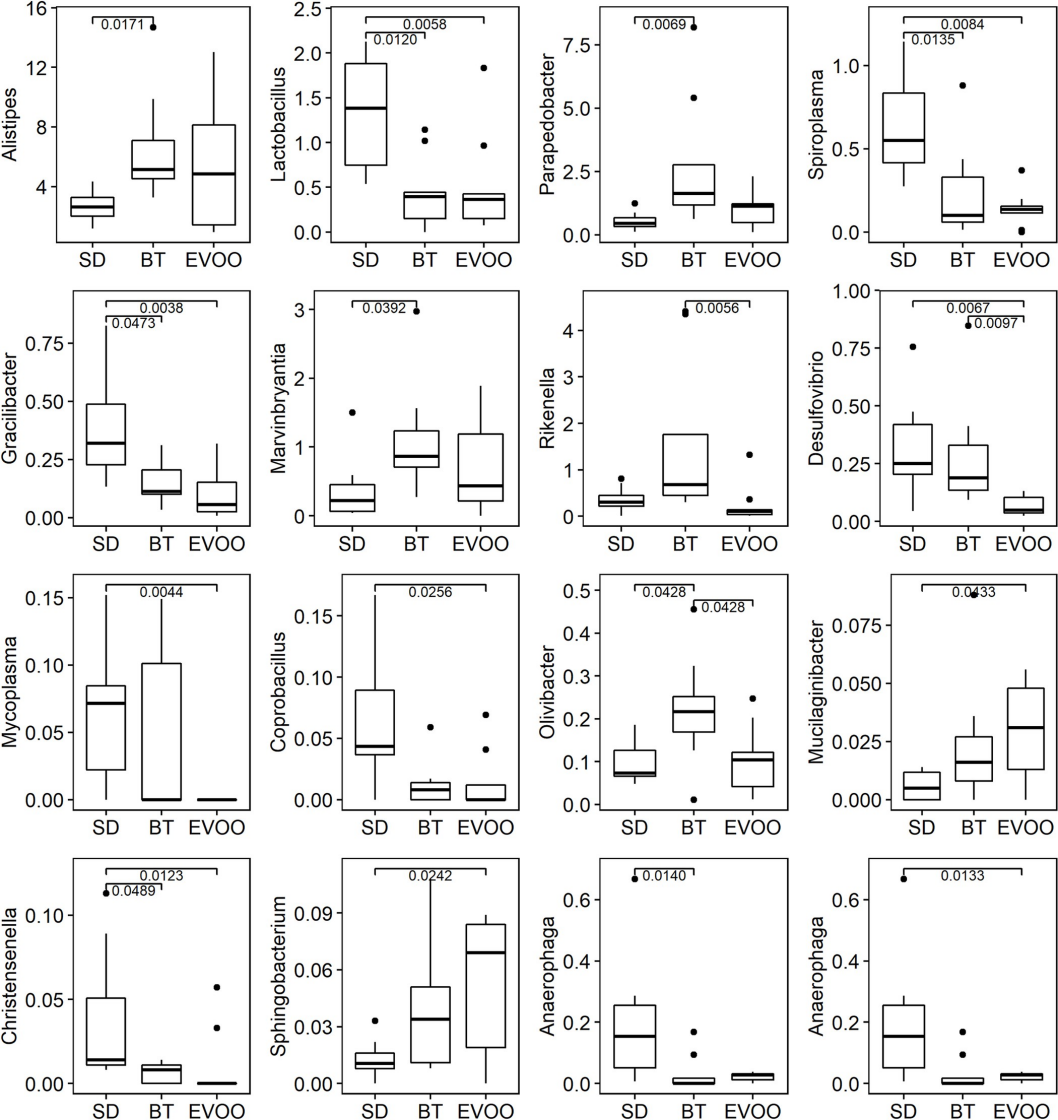
Box-plot representation of the percentage distribution of the sixteen genera with significant differences (p<0.05) among mice fed a diet enriched with butter (BT), virgin olive oil (EVOO) or standard chow (SD).

We also studied the percentages of the 227 genera obtained when studying the three diets. In this case, the Kruskal-Wallis test results indicated that sixteen of them have significant differences. Fig 4 shows the box-plots of all of these. Again, Table 4 shows the results found after applying a multiple linear regression analysis.

**Table 4:** Regression fits for each of the physiological variables studied using as independent variables those genera that show statistical differences in the percentage of sequences retrieved from faecal samples.

	BW		FI	WI		SBP	Total CHO	Glucose	HDL/LDL*	Leptin
	6w	12w	12w	6w	12w	12w	12w	12w	12w	12w
	0.39 0.0035	0.85 0.0028	0.77 0.0001	0.59 0.0006	0.72 0.0016	0.70 0.0001	0.31 0.0032	0.16 0.0425	0.86 0.0001	0.58 0.0001
*Alistipes*		-0.93 ±0.16 (0.0001)				4.94 ±1.13 (0.0004**)**		-5.64 ±2.63 (0.0425)		
*Lactobacillus*			0.33 ±0.11 (0.0074)							
*Parapedobacter*		2.49 ±0.36 (0.0000)							0.17 ±0.04 (0.0005)	
*Spiroplasma*		13.41 ±2.1 (0.0000)							0.83 ±0.22 (0.0015)	
*Gracillibacter*	8.67 ±3.04 (0.0090)	5.59 ±2.21 (0.0264)	-1.40 ±0.44 (0.0056)						1.13 ±0.3 (0.0024)	
*Marvinbryantia*		1.90 ±0.55 (0.0050)							0.44 ±0.09 (0.0002)	
*Rikenella*		0.81 ±0.31 (0.0217)	-0.28 ±0.06 (0.0001)			14.54 ±3.63 (0.0008)				
*Desulfovibrio*		-10.89 ±2.66 (0.0015)	0.879 ±0.26 (0.0041)		17.18 ±3.51 (0.0002)	54.82 ±18.14 (0.0070)				
*Mycoplasma*		-63.49 ±11.45 (0.0001)		31.61 ±10.18 (0.0059)	-42.06 ±13.78 (0.0081)				-6.05 1.32 (0.0004)	
*Coprobacillus*			4.63 ±1.73 (0.0156)		68.86 ±22.85 (0.0087)		-462.55 ±141.57 (0.0033)			
*Olivibacter*		11.33 ±3.90 (0.0132)			-15.59 ±6.78 (0.0362)					
*Mucilaginibacter*			13.89 ±3.14 (0.0003)	61.70 ±26.84 (0.0331)	161.71 ±45.54 (0.0029)	-617.78 ±222.42 (0.0120)			9.89 ±2.55 (0.0015)	-12526.4 ±5944.4 (0.0473)
*Christensenella*		-50.88 ±19.43 (0.0224)			-88.11 ±34.74 (0.0228)				-6.41 ±2.11 (0.0084)	
*Sphingobacterium*		-30.43 ±12.23 (0.0285)		38.94 ±17.49 (0.0383)					-0.86 ±0.38 (0.0426)	3697.60 ±874.440 (0.0004)
*Anaerophaga*		-10.78 ±3.16 (0.0052)							-1.62 ±0.67 (0.0301)	
*Marispirillum*	16.29 ±6.32 (0.0169)							-5.64 ±2.63 (0.0425)		

For each case, regression coefficient estimate, s.e. and p values are shown. R^2^ and p values of the model are also indicated under each physiological variable.

* indicates that logarithms of data have been used for the analysis. BW, body weight; FI, food intake; WI, water intake; SBP, systolic blood pressure; CHO, cholesterol.

**Fig 5:**
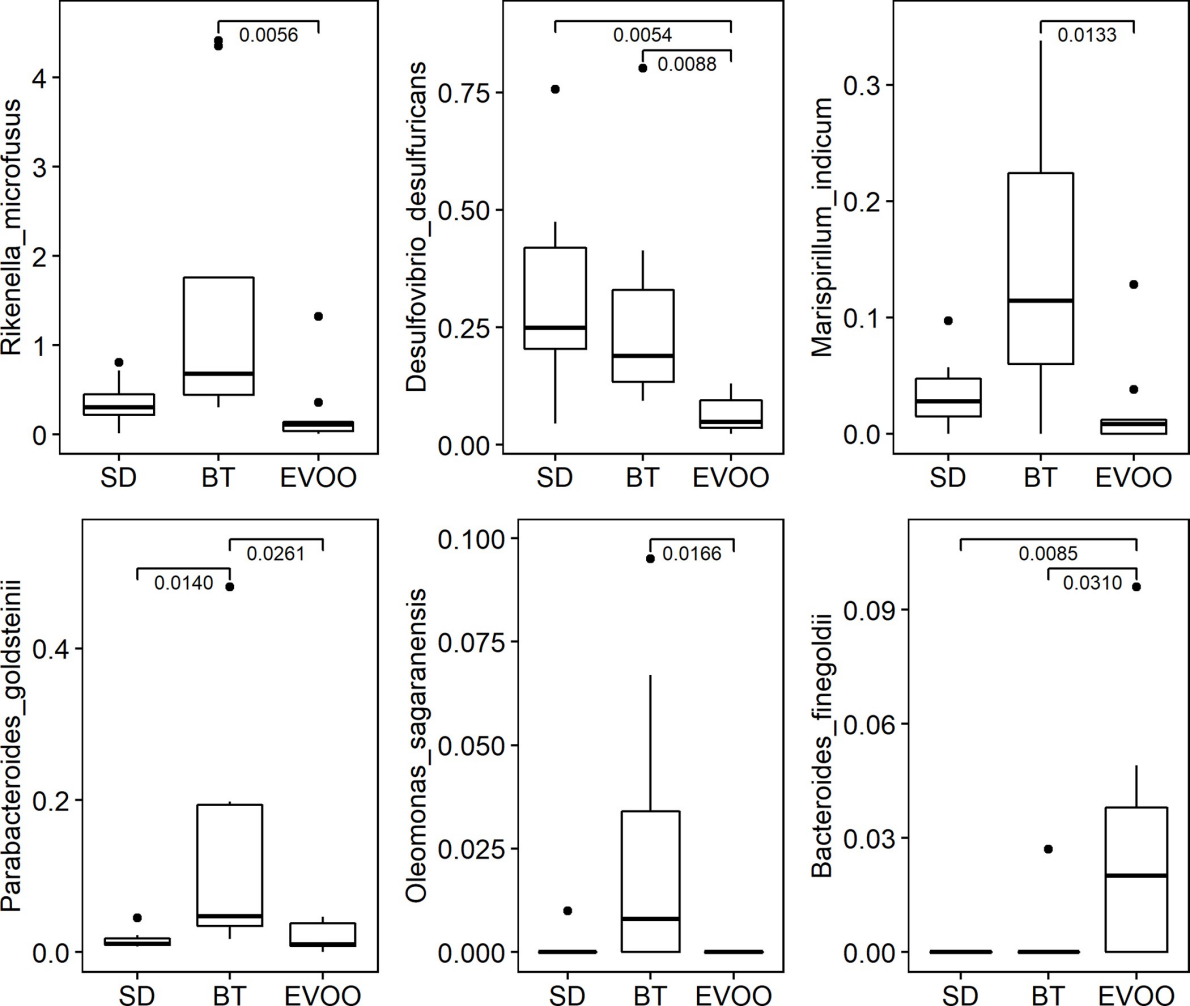
Box-plot representation of the percentage distribution of the six species with significant differences (p<0.05) between mice fed a diet enriched with butter (BT) and a diet enriched in virgin olive oil (EVOO). SD, standard chow.

Finally, sequences were also analysed at species level. When applying the Kruskal-Wallis test, 75 species showed significant differences among diets. Due to this high number of species, which made impossible a multiple regression analysis that included all of them, and since our main interest was to highlight the differences between the results obtained comparing the EVOO and the BT diets, we decided to focus our work on those cases in which the EVOO-BT pairwise comparisons showed statistically significant differences. This reduced the number of species to six, and their box-plot representation is shown in Fig 5. These six species were used as independent variables in the regression analysis fitted for each physiological variable studied, both at six and at 12 weeks (Table 5).

**Table 5:** Regression fits for each of the physiological variables studied using as independent variables those species that show significant differences between butter and virgin olive oil enriched diets.

	BW	DIU		FI	WI	SBP		Insulin	Leptin
	6w	6w	12w	12w	12w	6w	12w	12w	12w
	0.27 0.0065	0.25 0.0207	0.49 0.0032	0.27 0.0078	0.48 0.0070	0.25 0.0264	0.64 0.0001	0.16 0.0439	0.19 0.0340
*Desulfovibrio desulfuricans*			5.31 ±1.43 (0.0017)		9.79 ±3.89 (0.0216)		60.84 ±18.72 (0.0040)	1.26 ±0.59 (0.0439)	
*Parabacteroides goldsteinii*							106.38 ±41.31 (0.0181)		
*Bacteroides finegoldii*		25.06 ±11.51 (0.0206)	37.65 ±13.40 (0.0120)		96.14 ±36.42 (0.0167)	-484.62 200.43 (0.0264)			-11.51 ±5.09 (0.0340)
*Oleomonas sagaranensis*	81.78 ±27.34 (0.0065)			-31.32 10.76 (0.0079)	-78.03 ±32.91 (0.0291)		618.86 ±173.19 (0.0019)		

For each case, regression coefficient estimate, s.e. and p values are shown. R^2^ and p values of the model are also indicated under each physiological variable. BW, body weight; DIU, diuresis; FI, food intake; WI, water intake; SBP, systolic blood pressure.

## Discussion

In order to study the effect of different high fat diets on the intestinal microbiota, we have kept three groups of mice under standard chow alone or enriched with butter or virgin olive oil. This situation lasted for 12 weeks and, at the end of the experiment, differences between diets were observed and previously reported [19]. Briefly, in this 12-week study *Proteobacteria* in general and, specifically, *Desulfovibrionaceae* and *Desulfovibrio*, were significantly increased in the butter group, correlating with ghrelin in the case of the *phylum* and with blood pressure, water intake, diuresis and insulin in lower level taxa. EVOO showed a distinct behaviour with respect to butter in most cases and also occasionally with respect to standard diet. The link between diets, physiological parameters and the prevalence of certain taxa was clear but it was impossible to determine what was the cause and what was the consequence in this relationship. The fact of going back to study the situation in the middle of the experiment, six weeks earlier, can tell us how the intestinal microbial ecology of these mice was evolving in response to the new diets and it could open new hypothesis about the role of the different taxa in the interaction with the host.

In the present data, after six weeks of undergoing high fat diets, the only physiological variable with statistically significant differences was systolic blood pressure, which showed lower values in the EVOO group (p = 0.0039) but, notably, it did not show differences between BT and SD yet. Six weeks later, as indicated above [19], this scenario would be much more dramatic, and the BT group will show higher values, being significantly different from the SD diet as well. In this sense it is well known how damage inflicted by high fat diets is cumulative with time, including developing high blood pressure [23–25]. No other difference was detected at a physiological level, in contrast again with the results reported at 12 weeks, when the butter-fed group displayed significant higher values not only on blood pressure but also on body weight, among other hormonal and biochemical variables measured once the animal was sacrificed, like insulin, plasma triglycerides or total cholesterol [19].

When studying the microbial taxa obtained at the phylum level, *Proteobacteria* was detected as significantly higher in BT, maintaining this signification six weeks later. These bacteria are opportunistic, anaerobic-facultative microorganisms that will easily take profit of a change in the diet towards easily degradable foods like sugars or fats. Given the opportunity, they have a high growth-rate and can compete with slow-growing autochthonous groups like *Bacteroides*. At this point, *Proteobacteria* correlates with blood pressure, as it happens with many proteobacterial lower level taxa both in this work (see below) and at 12 weeks [19], although at 6 weeks the phylum has a negative correlation.

*Tenericutes* is also a phylum displaying significant differences at this time but they will not maintain this condition later on at the end of the experiment. This big bacterial group is characterized for being the only one displaying cholesterol in their membranes, which would make them good candidates to prevail in a butter enriched environment. Notably, this is not entirely the case at this level, since high numbers are detected also in the standard diet. Moreover, at family and genera level, pair-based signification in *Tenericutes*-framed taxa (*Spiroplasmataceae*, *Mycoplasmataceae* and related genera) always denotes higher values in the SD group. These results could be a reminiscence of the lactation period, where mice were fed with cholesterol-loaded milk, while in the EVOO group, polyphenols could have collaborated to eliminate these taxa earlier [26].

At family level, there are eleven different families showing pairwise differences between diets, belonging to the phyla of *Bacteroidetes* (*Rikenellaceae* and *Sphingobacteraceae*), *Firmicutes* (*Lactobacillaceae*, *Gracilibacteraceae*, *Christensenellaceae* and *Catabacteriaceae*), *Proteobacteria* (*Desulfovibrionaceae*, *Rhodospirillaceae* and *Acetobacteraceae*) and the already mentioned belonging to *Tenericutes* (*Spiroplasmataceae* and *Mycoplasmataceae*). The first result that called our attention was that the EVOO group showed statistically significant lower values in seven of these families and only in one case the levels reached in this group of mice were predominant, but still with low percentages throughout the three diets. Although this is not surprising, due to the antimicrobial properties of this fat [18, 21], the fact of reaching higher representation later on at the end of the experiment, with a clearly significant predominance in *Sutterellaceae* and *Erysipelotrichaceae* [19], increased the expectations in this sense. However, these two families did not show differences at six weeks yet. This could indicate that the bacterial adaptation to EVOO is not immediate, as we could also observe in a previous work on the fat-resilience of intestinal strains isolated from hosts fed different diets [21, 22].

*Lactobacillaceae* are lactic-acid bacteria (LAB) that are present within the intestinal microbiota in higher numbers during lactation but are proposed to decrease over time in the adult life [27, 28]. In our case, *Lactobacillaceae* and its correspondent genus, *Lactobacillus*, showed a significant decrease in mice fed the two high fat diets with respect to SD diet. This significant difference is lost at the end of the experiment [19], because numbers under SD diet also became finally lower at that point. Considering this data, it is possible that a chronic high fat diet could bring forward the LAB decrement in the mice lifespan with respect to a standard diet. *Lactobacillaceae* values measured at six weeks correlate with food intake, not only with the values registered at this timepoint but also with those at twelve weeks when this family is no longer significantly different between diets. This result is interesting because lactobacilli have been repeatedly associated to obesity [29], which could be related to food intake; in our case however, no relation with weight is found in spite of the continuous link of the family to FI.

As mentioned before, *Spiroplasmataceae* and *Mycoplasmataceae* have cholesterol in their membranes but cannot synthesize it [30, 31]. *Sphingobacteriaceae* is a similar case, being their name derived from the particular sphingolipids they contain in their walls. The presence of this lipid is rare in bacteria although it has been described in these taxa of the *Bacteroidetes* phylum and in certain alpha-*Proteobacteria*, as it is the case of *Acetobacteria* [32], also increased in the BT group. Sphingolipids can be obtained directly from the diet and the environment but they can also be synthesized from palmitic acid [33] and they have been proposed as important molecules in the establishment of the symbiosis and also as a source of possible pathogenesis when bacterial-produced sphingolipids enter host metabolic pathways impacting ceramide levels [34]. All these lipid-related families have some notable trait in common: they correlate with blood pressure at 12 weeks but they do not at 6 weeks, even though SBP is significantly different between groups at this time. Not only this, but also none of these families show significant differences at 12 weeks any longer. Therefore, it is quite remarkable that their relative levels in the intestines of young mice are somehow predicting the blood pressure values when these mice reach a more mature age. Moreover, a closely related alpha-proteobacterial family, *Rhodospirillaceae*, that shares the same taxonomical order with *Acetobacteraceae* but with no report of sphingolipids in its membranes, does not share this predictive behaviour and shows no correlation with blood pressure at 12 weeks. The same thing is observed with *Rikenellaceae*, also a *Bacteroidetes* family, but in a different order than *Sphingobacteriaceae* and with a different lipid composition.

It is worth noticing the case of *Gracilibacteraceae*, a *Clostridia* family (*Firmicutes*) that shows significantly higher values in the standard diet with respect to the two high fat diets but does not maintain this difference six weeks later. In the intermediate balance, the family values correlate with body weight and also, negatively, with food intake. What is more remarkable is that its values at six weeks correlate as well negatively with the food intake values at twelve weeks, even though *Gracilibacteraceae* does not present significant differences between diets at the end of the experiment. In spite of correlating with body weight at six weeks, the percentage of this family at six weeks does not correlate any longer with body weight at twelve weeks. In a long shot, this could be indicative of a self-regulatory mechanism in the food intake/body weight equilibrium involving this family.

Finally, the *Desulfovibrionaceae* family deserves a special mention, since it has shown very robust and repetitive implications throughout this research along several experiments [19, 20]. This family shows clear differences between the two high fat diet groups both at six (this work) and at twelve weeks [19, 20], with higher values in the butter group. This is also maintained at the level of genus (*Desulfovibrio*) and species (*D*. *desulfuricans*) during all the experimental period. At twelve weeks, these taxa correlated positively with insulin, WI, diuresis and blood pressure, all of them related to the metabolic syndrome [19]. At six weeks, the values obtained for these taxa do not correlate with any physiological variable, not even with blood pressure, which shows significant higher values in the butter group, as also *Desulfovibrionaceae* does. However, surprisingly, they clearly correlate with the values of blood pressure, insulin, FI and WI measured at the end of the experiment, all of them being metabolic syndrome markers, as well as negatively with body weight at this timepoint. Again, it is difficult to make a hypothesis on the cause/effect significance of this correlation but the evidence strongly supports an implication of the *Desulfovibrionaceae*/*Desulfovibrio* taxa in this pathology and its presence at an early age could clearly indicate more probabilities of suffering metabolic syndrome later on. In our previous report [19] we already proposed an important role of these taxa in blood pressure and cardiovascular diseases. These are sulphate-reducing bacteria that could use compounds found in butter, like chondroitin sulphate [35] or taurine-derived sulphur [36] as last electron acceptors under intestinal anaerobic conditions. Moreover, bacteria have been proposed to be involved in liver conjugation products like indosylsulphate [37], as well as in the production of trimethylamine N-oxide (TMAO) [38] which has been associated with atherosclerosis and several chronic diseases. In fact, TMAO was also associated with abundance of *Desulfovibrio* [39] and the levels of this bacterium were strongly correlated with the higher risk of adverse cardiovascular effects [40]. In the present work we show evidence indicating that this taxon could serve as a marker not only for metabolic syndrome in real time but also for a higher probability of suffering this condition in the future. Accordingly, our results support as well the possibility of a preventive effect of a diet high in virgin olive oil, considering the inhibition exerted by this fat on this bacterium.

Other than this, we describe additional lower level taxa showing statistical differences and/or correlations which were not included in the families mentioned above, like the genera *Marvinbryantia* (fam. *Lachnospiraceae*) and *Coprobacillus* (fam. *Erysipelothichaceae*), both of them framed in the phylum *Firmicutes*; the genus *Anaerophaga* (fam. *Marinilabiaceae*) and the species *Parabacteroides goldstenii* (fam. *Porphymonadaceae*) and *Bacteroides finegoldii* (fam. *Bacteroidaceae*), all of them in the *Bacteroidetes* phylum; and finally the alpha-*Proteobacteria Oleomonas sagarensis* (fam. *Acetobacteraceae*, order *Rhodospirillales*). The case of *B*. *finegoldii* is noteworthy because it is the only species significantly increased in the EVOO fed group with respect to both SD and BT diets and at the same time its presence inversely correlates with BP at six weeks, which is significantly decreased in the EVOO diet.

Globally, these are characterized by two main observations: first, while there are hardly any correlations between these bacterial taxa percentages and the physiological values measured at six weeks of the experiment, a high number of correlations are obtained between these same taxa percentages at six weeks and the physiological variables measured at twelve weeks. Second, almost all these correlations with the twelve-week physiological/metabolic values take place with the physiological variables (WI, FI, SBP, BW) and not that much with the metabolic values measured in plasma at the end of the experiment. This is different to the correlations found when using twelve-week microbiota to study the correlations [19], where links were found with both the physiological and metabolic values. This could give a hint on the type of mechanisms that relate microbiota and host.

The data obtained in this work, together with previous publications of ours [19, 20] and studies from other authors (see below), allow us to suggest the following scenario. In the case of the BT group, the butter supplementation produces an overload of cholesterol and other saturated fats onto the small intestine. This gives place to several immediate changes, mainly a) an increase of the intestinal permeability by stimulating proinflammatory signaling cascades and barrier disrupting cytokines while decreasing barrier-forming cytokines; b) a gradually more reduced environment, provoking an absolute lack of oxygen, even in areas where this circumstance is not complete; and c) a major induction of bile acid production, mainly hydrophobic, to cope with the excess of fat [41]. As a consequence of these three circumstances, a shift in the intestinal microbiota is prone to occur towards more strict anaerobic bile-resistant opportunistic bacteria which, like proteobacterial species, are able to grow rapidly profiting of the new ecological niche and to displace autochthonous species. The overgrowth of these strains will send several stress signals to the host, for instance, through microorganism or pathogen-associated molecular patterns that activate the host pattern-recognition receptors [42] and through bacterial specific molecules like Gram-negative lipopolysaccharides (LPS), typical of proteobacterial outer membranes, that are well known for triggering an immune response after being absorbed via the formation of chylomicrons [43], stimulating a TLR4-CD14–dependent proinflammatory response and producing endotoxemia [2, 44]. These and similar changes give place to a vicious cycle, increasingly debilitating the intestinal barrier. One species fitting this scheme is the proteobacteria *Desulfovibrio desulfuricans*, presenting LPS [45] and detected in higher percentage in the BT group in this work. This bacterium is a strict anaerobe, sulfate reducing bacteria (SRB), able to use sulfates and related oxidized molecules as electron acceptors instead of oxygen, and producing H_2_S as the reduced element. With an important role as H_2_-sink in the environment, SRB are not prominent in the intestines because they are overcome by other bacterial groups occupying this niche, as are methanogens or acetogens, that reduce CO_2_ instead. However, as it has been mentioned above, in the BT diet there is also a number of exogenous sulfate-conjugated metabolites already available in the butter [35, 36] and, in the host, part of the high amount of bile acids produced are 3α‑sulfated or 3β‑sulfated bile acids where the sulfate can be removed by many intestinal bacterial species [46]. All this makes the perfect environment for *D*. *desulfuricans* to thrive, producing H_2_S that not only can be toxic for the epithelial barrier [47] but can also modulate the hepatic bile acid metabolism by induction of the farnesoid X receptor (FXR) and inhibition of cholesterol 7 alpha-hydroxylase (CYP7A1), to produce more hydrophobic bile acids due to increased deoxycholic acid (DCA) and decreased β-muricholic acid (βMCA), eventually giving place to hepatic and biliary cholesterol overloading and even promoting gallstone formation [48]. Finally, *Desulfovibrio desulfuricans* was the first species where a system to produce trimethylamine (TMA) was detected and where TMA production was proven [49]. TMA is a well-studied microbial metabolite which is produced by bacteria carrying enzyme complexes like the choline utilization TMA lyase system (CutC/D) from nutrients like phosphatidylcholine and similar, that are present in high fat foods. TMA is further oxidized in the hepatic cells to produce trimethylamine N-oxide (TMAO), which is involved in the formation of atherosclerotic plaques, which are directly related to an increment in blood pressure [50]. All these events, specially the arteriosclerosis-related increment in BP, take time to develop and this could explain why *D*. *desulfuricans* measured at 6 weeks, when BP in the BT group is not elevated yet, does correlate with the values of this physiological parameter when measured six weeks later, at 12 weeks, when BP in mice fed the butter-enriched diet has the highest values.

On the other hand, the setup in the EVOO fed group is completely different. EVOO is also a high fat diet but it is enriched in monounsaturated fatty acids and it contains a minor compound fraction enriched in different polyphenols that is getting an important role in the prevention of the metabolic syndrome and hypertension [12, 13, 51]. The reductions in cyclooxygenase (COX)-2, metalloprotease, and interleukin 6 (IL6) are thought to be the leading mechanisms by which olive oil and its polyphenols exert anti-inflammatory activity [52]. One of the minor compounds, oleocanthal, has anti-inflammatory properties due to NSAID-like mechanisms of action [53] and EVOO has long proven to be implicated in the renin-angiotensin system [54, 55]. Results from in vitro studies have shown the capacity of polyphenols to increase the expression and/or production of numerous tight junction proteins and to reduce the release of several interleukins/cytokines [56]. We can clearly see this immediate effect in our results, since at six weeks, SBP in the EVOO group is lower than both the values in SD and BT and it does not present correlation with a robust bacterial percentage. Therefore, we think this quick BP decrement is due to the immediate vasodilator effects of the minor compounds [57]. In addition, studies on animal models clearly indicate the capacity of EVOO to modulate the intestinal microbiota due also to its antimicrobial properties [21, 58] and to the prebiotic function of its polyphenols whose low bioavailability allows them to reach the colonic microbiota highly unmodified [59]. In this way, EVOO is able to prevent the growth of deleterious bacteria and to enhance symbiont microbial groups [60]. Both beneficial effects can also be maintained over time since many of its active molecules are produced downstream the catabolism of precursors and because the systemic effects of the consequences of bacterial growth are noticed in a delayed manner [61]. In our specific case, EVOO is clearly preventing the growth of a blood pressure enhancer, *Desulfovibrio desulfuricans*, even in spite of the preference of this bacterium for a HFD environment, thanks to the antimicrobial effects of the olive oil minor polar compounds, as we showed in a previous publication [20]; EVOO will eventually also support the growth of other beneficial bacteria but this will be more evident after 12 weeks of experiment [19] and not at six weeks, indicating that the adaptation to grow with polyphenols might be slower than their antimicrobial action, as some other results already suggested [21].

In any case and overall, all these results are clearly suggestive of an implication of the diet-induced variations of the intestinal microbiota in the probability of developing altered physiological and biochemical responses later on in a subject’s lifespan.

## Materials and methods

### Animals

The procedures and experimental methodology have already been previously described [19]. The measurements reported here were performed at the mid-experiment balance, six weeks before the end of the experimental period that was already reported [19]. Briefly, twenty six (6 weeks old) male Swiss Webster ICR (CD-1) mice (Harlan Laboratories, 30.1 ± 0.55 g of initial weight) were fed *ad libitum* for three months a standard diet (SD; standard laboratory mice diet A04, 3% fat, Panlab, Barcelona, Spain) (n = 8) or one of two high fat diets (35% total energy, standard Panlab A04 chow supplemented with 20% fat) containing either butter (BT, n = 9) or extra virgin olive oil (EVOO, n = 9) respectively (Table 6). Fats were obtained and characterized as previously described [19]. All experimental procedures were reviewed and approved by the Bioethics Committee of the University of Jaén and performed in accordance with the European Communities Council Directive 86/609/EEC. Animals were individually housed in metabolic cages twenty-four hours before the six weeks of the experimental period. Food intake (FI), water intake (WI), diuresis (DIU), body weight (BW) and systolic blood pressure (SBP) were then measured and additionally, faecal samples were obtained. SBP was monitored as previously described [19, 62]. Animals were kept in cages during six more weeks, the procedure was repeated at the end of the experimental period and blood samples were obtained as described and already reported [19], obtaining the values of insulin, fasting glucose, triglycerides, total cholesterol, HDL, leptin and ghrelin [62–64]. All analyses were performed according to the manufacturer’s protocols.

**Table 6:** Nutrient composition and energy content of standard and high fat diets enriched with extra virgin olive oil and butter.

Diet	SD		EVOO		BT	
	g/100 g	% energy	g/100 g	% energy	g/100 g	% energy
**Protein**	16.5	20	16.5	14	16.5	14
**Carbohydrates**	60	72	55	48	55	48
**Fat**	3	8	20	38	20	38
**Total energy (kJ/g)**	14.2		19.6		19.6	

### Microbial biodiversity

Total faecal DNA obtained after 6 weeks was extracted using QIAamp^©^ DNA Stool Kit as it was already described for samples after 12 weeks [19]. Twenty six DNA samples, corresponding to eight mice under a standard diet, nine mice fed high butter and nine fed a high extra virgin olive oil diet, were sequenced (Lifesequencing, Valencia, Spain) as previously described [19]. Briefly, twenty six libraries were constructed, quantified, filtered to meet a Q20 threshold and checked for quimeras. The resulting sequences were assigned to different taxonomic levels using the Ribosomal Database Project Classifier. Rarefaction curves were obtained for each sample and taxonomical levels were analysed in order to confirm they had reached the plato and no more taxonomical groups were expected to be found if sequencing were increased.

### Statistical studies

To test the hypothesis of equality between central tendency measures according to the type of diet, the ANOVA test or the Kruskal-Wallis test were used, depending on whether or not the initial hypotheses were verified. In all cases a significance level of 5% was used. Pairwise comparisons were subsequently carried out wherever overall significant differences were detected. Afterwards, multiple regression models were fitted by backward elimination to explain physiological or biochemical variables according to those that showed significant differences between diets. All computations were made using SPSS 24 IBM (Armonk, NY, USA), R 4.0.2 (Auckland, New Zealand), and Gretl 2018c (San Diego, CA, USA).
